# A Systematic Review of the Magnetic Resonance Imaging Literature Investigating the Deficit Syndrome in Schizophrenia

**DOI:** 10.1093/schizbullopen/sgag009

**Published:** 2026-04-01

**Authors:** Matheus Teles G de Araujo, Jordan Sloan Larson, James Edward Bryant, Nina Vanessa Kraguljac

**Affiliations:** Department of Psychology, University of Alabama at Birmingham, Birmingham, AL 35233, United States; Department of Psychiatry and Behavioral Health, The Ohio State University, Columbus, OH 43210, United States; Department of Psychiatry and Behavioral Health, The Ohio State University, Columbus, OH 43210, United States; Department of Psychiatry and Behavioral Health, The Ohio State University, Columbus, OH 43210, United States; Department of Psychiatry and Behavioral Health, The Ohio State University, Columbus, OH 43210, United States

**Keywords:** deficit syndrome, schizophrenia, imaging

## Abstract

**Background and Hypotheses:**

Heterogeneity in schizophrenia is significant but remains poorly understood. Treatment strategies have not evolved beyond one-size-fits-all approaches, often delaying the discovery of effective treatments and better outcomes. The deficit syndrome was proposed as a more homogenous subtype of the illness that is characterized by primary and enduring negative symptoms. We reviewed Magnetic Resonance Imaging (MRI) studies examining brain structure, function, and neurometabolism in deficit syndrome with the goal of identifying unique brain signatures for this subtype of schizophrenia.

**Study Design:**

Systematic Review of MRI studies published between 1999 and 2024 on the deficit syndrome.

**Study Results:**

Potentially unique features of the deficit syndrome include nucleus accumbens and cerebellar gray matter volume deficits, reduced uncinate fasciculus fractional anisotropy, fronto-parietal and default mode network dysconnectivity, altered global efficiency, and shortest path-length in brain network organization. Further, abnormalities in cerebellar culmen volume and cerebellar spontaneous activation may differ only in degree between patient groups. Many findings warrant replication and must be interpreted with caution.

**Conclusions:**

The deficit syndrome is hypothesized to be different from other forms of schizophrenia and more homogenous in its pathophysiology. Magnetic Resonance Imaging studies examining neurobiological group differences show conflicting results; some report that the deficit syndrome may be distinct, while others find that brain pathology is more severe or shared across subtypes. No MRI studies have directly tested if inter-individual heterogeneity in brain pathology is lower in the deficit syndrome, which would be necessary to determine if this clinically more homogenous subtype is also neurobiologically more homogenous.

## Introduction

Clinical and neurobiological heterogeneity in schizophrenia is significant but remains poorly understood.^1–6^ Because of this translational gap, treatment strategies have not evolved beyond one-size-fits-all/ trial-and-error approaches, which too often lead to delays in finding effective treatments and in poor overall outcomes. To bridge this gap, it has been proposed that clinical characteristics can be leveraged to identify more similar subgroups of patients within this complex syndrome. These subgroups share disease mechanisms and trajectories, show similar patterns of response to specific pharmacological approaches, and have similar overall outcomes.

Perhaps the most prominent example in this context is the deficit syndrome,^3^^,^^7^^,^^8^ which was proposed by Carpenter *et al.* in the 1980’s as a subtype of the illness that is characterized by enduring and primary negative symptoms that are present even during periods of clinical stability.^8^ It is more prevalent among males,^9^ and carries a greater likelihood of summer births,^10^ delay in reaching developmental milestones,^11–16^ poorer premorbid functioning,^17^ and a more insidious onset of symptoms.^18^ These patients are also less likely to respond to conventional antipsychotic medications^7^^,^^12^^,^^19–21^ and endure a greater overall disease burden^22^ compared to those without persistent negative symptoms.

Together, this suggests that the deficit syndrome may be a distinct disorder within the schizophrenia syndrome, rather than just a more severe presentation of the same illness. In that case, this subtype should also display specific brain characteristics that differentiate it from the non-deficit form of the illness. The first neuroimaging study used Positron Emission Tomography (PET) to assess neurometabolic changes in 12 unmedicated patients with schizophrenia.^23^ They reported that hypometabolism in frontal, parietal, and thalamic regions was only found in patients suffering from the deficit syndrome, while hypometabolism in limbic regions was a shared feature among the overall group. Based on this seminal study, multiple neuroimaging investigations have been conducted to further characterize neurobiological differences between patients who do or do not meet the clinical criteria for the deficit syndrome. However, to our knowledge, no systematic review synthesizing the existing Magnetic Resonance Imaging (MRI) literature across different modalities exists to date.

Here, we performed a systematic review of case–control studies examining brain structural, functional, and neurometabolic signatures in the deficit syndrome with the overarching goal to identify shared and distinct neurobiological signatures within the schizophrenia syndrome.

## Methods

### Inclusion and Exclusion Criteria

We included MRI studies published between 1999 and 2024 (last search conducted on August 1, 2024) in individuals with schizophrenia spectrum disorders, either meeting or not meeting criteria of the deficit syndrome, and a healthy control comparison group. Our search criteria excluded articles published in languages other than English, as well as review papers and meta-analyses. We also excluded studies that did not have an MRI component, did PET imaging or electroencephalogram (EEG), studies that did not explicitly focus on the deficit syndrome, and those with a sample size smaller than 10 participants per group. Additionally, we did not consider research that expressly included subjects with comorbid substance use disorders, neurological or genetic conditions, or intellectual disabilities.

### Literature Search

M.T. and N.V.K. conducted a comprehensive literature search in PubMed for studies related to the psychosis spectrum using the following key words: (“deficit features” OR “Deficit syndrome” OR “deficit schizophrenia”) AND (gray matter OR morphometry OR VBM OR volume OR white matter OR DTI OR magnetic resonance spectroscopy OR MRS OR functional MRI OR resting-state). We also searched for additional relevant publications using the reference lists of the manuscripts included in this systematic review.

### Study Selection

M.T. and N.V.K. screened the titles and abstracts obtained from the search to identify potentially eligible studies for full-text review. Both authors applied the eligibility criteria, resulting in a consensus-driven list of full-text articles deemed eligible. Full-text articles were downloaded or requested from the university library and assessed for eligibility.

### Data Extraction

We extracted the following data from each study: name of first author, year of publication, number of participants per diagnostic category, mean age per diagnostic group, percent of male participants, how the deficit was operationally defined, illness duration, medication status, regions of interest in the brain, analysis method, magnetic field strength, measures acquired, main study outcomes and associations between brain measures and negative symptom severity. For studies using resting-state functional imaging, we also extracted whether participants’ eyes were open or closed. For Magnetic Resonance Spectroscopy (MRS) studies, we also extracted neurometabolites of interest and MRS sequence parameters. Differences in hemispheric asymmetry reported in the eligible publications were not included in this review due to the limitations of widely used threshold-dependent methods.^24^

## Results

Our initial search yielded 71 potentially relevant studies. Following the study selection process outlined in Figure 1, we included a total of 14 studies on gray matter, 9 on white matter, 8 on resting-state imaging, 1 on task activation, and 2 publications on MRS in this systematic review. Whenever applicable, the following sections are organized as follows: overall information, findings replicated in at least 2 independent samples, findings unique to the deficit syndrome group, findings unique to the non-deficit syndrome group, shared features, evidence for the deficit syndrome as a more severe form of schizophrenia, and contradictory reports.

### Structural MRI Studies

#### Gray Matter

Fourteen reports assessing gray matter indices met eligibility criteria for inclusion in the review. Table 1 summarizes extracted data. Image analysis techniques were variable, areas of interest differed across studies, and measures to quantify gray matter pathology included volumetric, cortical thickness, and cortical surface area indices. Though not necessarily explicitly stated, several reports appear to have overlapping samples: 1.,^25^^,^^26^ 2.,^27^^,^^28^ 3.,^29^^,^^30^ and 4.^31^^,^^32^

**Table 1:** Structural MRI, Studies Examining Gray Matter

**Author**	**Year**	***n* HC/DS/NDS**	**Age HC/DS/NDS; mean (SD)**	**Sex HC/DS/NDS; % male**	**Findings with direct comparisons between HC, DS, and NDS**	**Findings with pairwise comparisons only**	**Associations with symptoms**	
Li et al.^27^	2023	38/33/39	46.1 (9.1)/49.1 (7.7)/45.9 (5.4)	100/100/100	DS = NDS < HC in hippocampal subfield volumes		N/A	
Hepdurgun et al.^38^	2021	18/13/26	Median (IQR): 36 (9)/34 (13)/36 (12)	61/77/77		NDS = DS > HC at baseline, trajectories over 5 years differ between the groups	N/A	
Lei et al.^26^	2019	32/14/20	21.6 (4.7)/21.8 (5.4)/22.2 (6.7)	71/71/60	DS < NDS = HC in GMV in the cerebellar culmen		Negative correlation between GMV in the cerebellar culmen and negative symptoms severity in patients	
Xie et al.^28^	2019	41/33/41	45.8 (9.5)/49 (7.7)/45.7 (6.6)	100/100/100	DS = NDS = HC in total surface area (DS = NDS) < HC in cortical thickness in inferior frontal gyrus and superior temporal gyrus		Mean cortical thickness of the inferior frontal gyrus was positively correlated with cognitive flexibility and visuospatial memory performance in DS and NDS.	
Takahashi et al.^29^	2017	59/38/37	26.1 (5.1)/27.1 (7.5)/27.1 (6.2)	47/32/57	DS < NDS = HC in IA length (DS = NDS) < HC in olfactory sulcus depth		None	
De Rossi et al.^36^	2016	22/22/22	38.3 (11.5)/39.2 (11.1)/38.3 (11)	77/77/77	DS < (NDS = HC) in nucleus accumbens volume (DS = NDS) < HC in thalamus volume		Age and duration of illness were significantly associated with lower volume of the accumbens in DS but not in NDS	
Lei et al.^25^	2015	88/44/44	22.6 (6.3)/22.9 (6.9)/23.2 (6.9)	56/56/56	DS < NDS < HC in GMV in the cerebellar culmen DS < (NDS = HC) in GMV in the insula		none	
Takayanagi et al.^30^	2013	82/18/30	43.7 (12.7)/35.9 (11.8)/44.3 (10.5)	48/83/66	DS = NDS = HC in surface area and cortical thickness		NA	
Voineskos et al.^33^	2013	79/18/59	43 (14)/49 (15)/43 (14)	60/77/64	DS = NDS = HC in cortical surface area and subcortical volumes (DS = NDS) < HC in cortical thickness in the OFC, middle temporal gyrus, temporal pole, DLPFC, parietal operculum, parahippocampal gyrus, superior temporal gyrus, and insula		N/A	
Özdemir et al.^34^	2012	17/11/18	33.8 (10.1)/32.4 (8.2)/40.8 (12.3)	52/63/50	(DS = NDS) < HC in total GMV		N/A	
Fischer et al.^35^	2012	28/20/36	36.0 (6.6)/40.1 (7.2)/38.4 (7.9)	82/85/86	DS = NDS = HC gray matter volume in the dorsolateral prefrontal-basal ganglia-thalamocortical circuit DS < NDS = HC superior frontal and inferior frontal gyrus, middle temporal gyrus DS = NDS < HC in amygdala-hippocampal complex		N/A	
Arango et al.^37^	2008	66/19/66	Male HC: 33 (6.8)/Male patients: 38.4 (7.1); Females HC: 35.3 (7.8)/Female patients: 40.3 (6.3)	52/89/N/A	DS = NDS = HC in total cranial volume (DS = NDS) < HC in total brain volume HC < (DS = NDS) in sulcal CSF volume DS < NDS = HC in ventricular volume		N/A	
Galderisi et al.^31^	2008	31/34/32	34.4 (8.3)/35.8 (7.4)/34.2 (8.1)	67/73/81	(DS = NDS) < HC in GMV in DLPFC and temporal lobe DS = NDS = HC in GMV in hippocampi, caudate nuclei, globus pallidi, and putamen volumes HC = DS < NDS lateral ventricular volumes		N/A	
Quarantelli et al.^32^	2002	25/14/14	Age range 18-50/37.1 (8.4)/34.8 (8.5)	76/92/92	(DS = NDS) < HC in total fractional GMV (DS = NDS) < HC in GMV in frontal and temporal lobes (DS = HC) < NDS in ventricular volume HC < (DS = NDS) in intracranial CSF		N/A	
Li et al.^27^	2023	SDS^a^	Chronic	Medicated	Hippocampal subfields	ROI	Hippocampal subfield volumes	3
Hepdurgun et al.^38^	2021	SDS	Chronic	Medicated	Lateral ventricles	Free Surfer	Ventricular volume	3
Lei et al.^26^	2019	SDS	FE	Medication naive	Whole brain + ROI analysis	VBM	GMV	3
Xie et al.^28^	2019	SDS^a^	Chronic	Medicated	Whole brain	CIVET	Cortical thickness, surface area	3
Takahashi et al.^29^	2017	PDS	Chronic	Medicated	Interthalamic adhesion, OFC	Manual ROI’s	IA length, OFC pattern distribution, OFC depth, IOS/POS count	1.5
De Rossi et al.^36^	2016	SDS	Chronic	Medicated	Nucleus accumbens, thalamus	Free Surfer	GMV	3
Lei et al.^25^	2015	SDS	FE	Medication naive	Cerebellar culmen, insula	ROI	GMV	3
Takayanagi et al.^30^	2013	SDS	Chronic	Medicated	ACC	ROI	GMV, cortical thickness, surface area	1.5
Voineskos et al.^33^	2013	PDS	Chronic	Medicated	Whole brain	VBM, FSL	Cortical thickness, surface area, GMV	1.5
Özdemir et al.^34^	2012	SDS	Chronic	Medicated	Whole Brain	VBM	GMV	1.5
Fischer et al.^35^	2012	SDS	Chronic	Medicated	DLPFC, IPC, thalamus, caudate, orbital frontal, inferior frontal, superior frontal gyri, superior, middle temporal gyri, amygdala-hippocampus complex	VBM	GMV	1.5
Arango et al.^37^	2008	SDS	Chronic	Medicated	Whole brain	MEASURE	Total cranial volume, total brain volume, sulcal CSF volume, ventricular volume	1.5
Galderisi et al.^31^	2008	SDS	Chronic	Medicated	Lateral ventricles, DLPFC, cingulate gyrus, temporal lobe, hippocampus, caudate nucleus, putamen, globus pallidus	Automated ROI analysis/Manual tracing	GMV, ventricular volume	1.5
Quarantelli et al.^32^	2002	SDS	Chronic	Medicated	Whole brain	Manual tracing/Automated method	GMV, ventricular volume, intracranial CSF volume	N/A
**Structural MRI, studies examining white matter**								
**Author**	**Year**	***n* HC/DS/ NDS**	**Age HC/DS/NDS; mean (SD)**		**Sex HC/DS/NDS; % male**	**Findings with direct comparisons between HC, DS, and NDS**	**Associations with symptoms**	
Türk et al.^46^	2023	22/15/25	36.1 (12.3) / 37.5 (10.6) / 40 (13.1)		63/53/68	(DS = NDS) < HC in FA except for cuneus where DS < NDS = HC (DS = NDS) > HC in MD (DS = NDS) > HC in RD	N/A	
Podwalski et al.^40^	2022	36/26/42	37.4 (7.8) / 38.4 (6.5) / 38.6 (7.1)		41/73/42	DS = NDS < HC in FA in the superior longitudinal fasciculus	None	
Podwalski et al.^39^	2021	30/15/40	37.3 (8.2) / 39.8 (6.1) / 38.8 (7.2)		46/73/45	DS < (NDS = HC) in FA in the splenium of the CC DS = NDS > HC in MD in the splenium of the CC	None	
Tan et al.^41^	2020	111/45/133	33.3 (10)/34.5 (8.8)/33.4 (9.6)		59/66/67	DS = NDS < HC in FA in the body of the CC DS < NDS < HC in FA in the posterior thalamic radiation		
Lei et al.^25^	2015	41/33/42	23.5 (7.3)/22.3 (6.9)/23.4 (7.1)		58/66/59	DS < (HC = NDS) in WMV in extra-nuclear white matter volume (DS = NDS) < HC in FA in body of CC, posterior superior longitudinal fasciculus	N/A	
Spalletta et al.^43^	2015	21/21/21	33.8 (1.5)/33.8 (12.2)/34.1 (12.1)		90/90/90	NDS < DS < HC in FA in cerebellum, occipital lobe, inferior fronto-occipital fasciculus, forceps major (DS = NDS) < HC in FA in anterior, superior and posterior corona radiata, forceps minor, external capsule, cerebral peduncle, body and genu of CC (HC = NDS) > DS in AD in postcentral area (DS = NDS) < HC in AD in cerebellum NDS > (DS = HC) in RD in superior corona radiata, internal and external capsules, temporal lobe NDS > DS > HC in RD in frontoparietal areas, cerebellum DS > NDS > HC in RD in forceps minor (NDS = DS) > HC in RD in superior and inferior longitudinal fasciculus, posterior corona radiata, occipital lobe, body and genu of CC, interior fronto-occipital fasciculus, forceps major NDS > DS > HC in MD in occipital lobe	N/A	
Voineskos et al.^33^	2013	79/18/59	43 (14)/49 (15)/43 (14)		60/77/64	DS < NDS = HC in FA in the uncinate fasciculus DS > NDS = HC in MD in the uncinate fasciculus and inferior longitudinal fasciculus	PDS score positive correlates with MD in the uncinate fasciculus	
Kitis et al.^44^	2012	17/11/18	33.8 (10.1)/32.4 (8.2)/40.8 (12.3)		52/63/50	DS < NDS = HC in FA in the uncinate fasciculus	None	
Rowland et al.^45^	2009	11/10/10	37 (10)/43 (6)/40 (6)		72/80/80	DS < NDS = HC in FA in the right superior longitudinal fasciculus DS = NDS < HC in FA in middle frontal white matter DS = NDS = HC in WMV	None	
Türk et al.^46^	2023	SDS	Chronic	Medicated	Whole brain	ROI analysis	FA, MD, RD	1.5
Podwalski et al.^40^	2022	PDS	Chronic	Medicated	Superior longitudinal fasciculus	ROI analysis	FA	3
Podwalski et al.^39^	2021	PDS	Chronic	Medicated	Corpus callosum	ROI analysis	FA, MD	3
Tan et al.^41^	2020	PDS	Chronic	Medicated	Whole brain	TBSS	FA	3
Lei et al.^25^	2015	SDS	FE	93% medication naïve	Whole brain	TBSS	WMV, FA	3
Spalletta et al.^43^	2015	SDS	Chronic	Medicated	Whole brain	TBSS	FA, AD, RD, MD	3
Voineskos et al.^33^	2013	PDS	Chronic	Medicated	Uncinate fasciculi, inferior occipitofrontal fasciculus, cingulum bundle, inferior longitudinal fasciculus, arcuate fasciculus, and genu and splenium of the corpus callosum	Deterministic tractography, ROI analysis	FA, MD	1.5
Kitis et al.^44^	2012	SDS^b^	Chronic	Medicated	Uncinate fasciculi	TBSS, ROI analysis	FA	1.5
Rowland et al.^45^	2009	SDS	Chronic	Medicated	Superior longitudinal fasciculus, middle frontal and parietal white matter	Deterministic tractography, ROI analysis	FA, WMV	3

^a^Chinese version.

ACC = anterior cingulate cortex; CSF = cerebrospinal fluid; DLPFC = dorsolateral prefrontal cortex; DS = deficit syndrome schizophrenia; FE = first episode patients; FSL = FMRIB Software Library; GMV = gray matter volume; HC = healthy controls; IOS = intermediate orbital sulcus; POS = posterior orbital sulcus; IPC = inferior parietal cortex; IQ = intelligence quotient; IQR = interquartile range; LGI = local gyrification index; MEASURE = image analysis software; N/A = not applicable; NDS = Non-deficit syndrome schizophrenia; OFC = orbitofrontal cortex; PANSS = Positive and Negative Syndrome Scale; PDS = Proxy for the Deficit Syndrome; PFC = prefrontal cortex; ROI = region of interest; SD = standard deviation; SDS = Schedule for the Deficit Syndrome; SZ = patients with schizophrenia; VBM = voxel-based morphometry; WMV = white matter volume.

^b^Turkish version.

AD = axial diffusivity; CC = corpus callosum; DS = deficit syndrome schizophrenia; FA = fractional anisotropy; FE = first episode patients; HC = healthy controls; MD = mean diffusivity; N/A = not applicable; NDS = Non-deficit syndrome schizophrenia; PANSS = Positive and Negative Syndrome Scale; PDS = Proxy for the Deficit Syndrome; PFC = prefrontal cortex; PLIC = posterior limb of the internal capsule; PTR = posterior thalamic radiation; RD = radial diffusivity; ROI region of interest; SD = standard deviation; SDS = Schedule for the Deficit Syndrome; Sz = schizophrenia patients; TBSS = tract-based spatial statistics; VBM = voxel-based morphometry; WMV = white matter volume.

Findings that were replicated in at least 2 independent samples include similar cortical surface area measures between patient groups and healthy controls (HC),^28^^,^^30^^,^^33^ gray matter volume reductions^32^^,^^34^ and cortical thinning in fronto-temporal areas as shared features in both groups.^28^^,^^33^ Two studies reported hippocampus [subfield] volume reductions^27^^,^^35^ in both subtypes of the illness, others found only non-significant volume reductions in both patient groups,^33^ and one did not find hippocampus volume alterations in either patient group.^31^

Other findings have not been independently replicated. One study reported gray matter volume alterations in the nucleus accumbens,^36^ and cerebellar culmen,^26^ were unique to the deficit syndrome. Others reported reduced interthalamic adhesion length, a neurodevelopmental marker, only in the deficit syndrome.^29^

The idea that the deficit syndrome may be a more severe form of the illness rather than a separate disease entity is supported by 1 study reporting a greater reduction in cerebellar culmen volumes in the deficit syndrome compared to the non-deficit form of the illness.^26^ Others find that the extent of pathology, like thalamus volume deficits^36^ and olfactory sulcus depth reduction,^29^ does not differ in the 2 subtypes.

Finally, 2 studies investigating gray matter features directly contradict each other. Insula volume reduction was found to either only be present in the deficit syndrome,^25^ or a shared feature.^33^ Difference in findings could be due to distinctions in illness chronicity and age, that is, 1^25^ group had younger first-episode psychosis patients while the other^33^ group had older chronically ill and medicated patients. Additionally, ventricle enlargement was found to be most prominent in the deficit syndrome^37^ in 1 study, while another reported it to be a unique feature of the non-deficit form of the illness,^31^ and a third reported it as a shared feature between the 2 subgroups,^38^ albeit with different trajectories in progression 5 years later. While clinical and demographic characteristics were similar in these studies, the different findings may be associated with distinctions in analysis methods, which included manual tracing,^31^ Free Surfer,^38^ and MEASURE.^37^

#### White Matter

Ten reports assessing white matter indices met eligibility criteria for inclusion in the review. Table 2 summarizes the extracted data. The majority of studies used diffusion tensor imaging to quantify white matter indices; only 2 assessed volumetric measures. Analyses either focused whole brain measures or on one or more a priori selected specific white matter tracts. Two reports appear to have overlapping samples.^39^^,^^40^

**Table 2:** Functional MRI Studies, Resting-State fMRI

**Author**	**Year**	***n* HC/DS/NDS**	**Findings with direct comparisons between HC, DS, and NDS**				**Findings with pairwise comparisons only**		**Associations with symptoms**		
Teles et al.^47^	2024	72/18/43	DS < (HC = NDS) in global efficiency DS < (HC = NDS) in global local efficiency DS > (HC = NDS) in global shortest path length DS = HC = NDS in global clustering coefficient, synchronization, and small-worldness						None		
King et al.^48^	2022	73/25/53	HC = NDS > DS in FC in the frontoparietal control network A DS = NDS = HC (DS = NDS) in FC in the frontoparietal control network B						None		
Fan et al.^49^	2022	40/33/41	(DS = NDS) < HC in intramodule connectivity in VN, SMN DS < NDS < HC in intramodule connectivity in SN (DS = NDS) > HC in intermodule connectivity between SMN and DMN DS > (NDS = HC) in intermodule connectivity between SMN and FPN NDS < DS < HC in intermodule connectivity between SMN and VN (DS = NDS) < HC in normalized within-module degree of postcentral gyrus, lingual gyrus (NDS = HC) < DS in normalized within-module degree of superior temporal gyrus (NDS = HC) > DS in normalized within-module degree of precentral gyrus, anterior cingulate gyrus (DS = NDS) > HC in normalized within-module degree of cerebellar vermis DS > NDS > HC in normalized within-module degree of precuneus (DS = NDS) > HC in patterns of participation coefficient of 24 regions in frontal and parietal lobes DS > (NDS = HC) in participation coefficient of postcentral gyrus NDS > (DS = HC) in participation coefficient of supplementary motor area and pallidum Intramodule connectivity of SN, intermodule connectivity of SMN-VN, and normalized within-module degree of precuneus were all able to distinguish DS, NDS, and HC from one another						N/A		
Zhou et al.^52^	2021	113/58/93	(DS = NDS) < HC in FC between LNAcc and middle cingulate gyrus (DS = NDS) < HC in FC between RNAcc and middle frontal gyrus (DS = NDS) > HC in FC between bilateral NAcc and lingual gyrus						FC between LNAcc and superior temporal gyrus was negatively correlated to BPRS total in DS. FC between LNAcc and lingual gyrus was positively correlated with BPRS total in DS. FC between LNAcc and middle frontal gyrus was positively correlated with DVT score in DS. FC between LNAcc and middle cingulate gyrus was negatively correlated with BPRS total and positively correlated with COWAT scores in NDS. FC between RNAcc and lingual gyrus was negatively correlated with spatial processing test score and positively correlated with Stroop words test score in NDS.		
Zhou et al.^51^	2019	40/33/41	(NDS = DS) < HC in fALFF in sensorimotor regions, visual cortex, and frontoparietal pathway (DS = NDS) > HC in fALFF in the precuneus and posterior cingulate cortex NDS > DS = HC in fALFF in the hippocampus						In the NDS group, fALFF value of the insula was negatively correlated with speech fluency		
Zhou et al.^50^	2019	38/37/38	(DS = NDS) > HC in DMN activity in angular gyrus and calcarine sulcus (DS = NDS) < HC in DMN activity in middle frontal gyrus DS > (NDS = HC) in DMN activity in PCC						DS TMT-B scores correlated positively with neural activity from the precuneus. The Stroop color scores correlated negatively with the activity of the PCC, and spatial processing scores correlated positively with the activity of the PHP/ HIP in DS. NDS TMT-B scores correlated independently with neural activity from the MFG and CAL. WAIS-RC scores correlated negatively with neural activity from CAL in NDS.		
Yu et al.^53^	2017	40/33/41	DS = NDS = HC in small-worldness and normalized clustering coefficient (DS = NDS) < HC in clustering coefficient and local efficiency DS < (NDS = HC) in characteristic path length and normalized global characteristic path length DS > (NDS = HC) in global efficiency						IN DS BPRS, total scores correlated positively with global efficiency and negatively with characteristic path length		
Li et al.^54^	2017	4241/42	DS > NDS > HC in ALFF in putamen NDS < DS < HC in ALFF in cerebellum posterior lobe (DS = NDS) > HC in ALFF in fusiform gyrus				DS > HC in ALFF in insula NDS = HC in ALFF in insula NDS < HC in ALFF in middle temporal gyrus		ALFF in the putamen was positively associated with PANSS-NS and PANSS-WIT in DS, but not in NDS. ALFF in the putamen was positively associated with PANSS-PS and PANSS-EXC/ACT in NDS, but not in DS. ALFF in the cerebellum posterior lobe negatively correlated with PANSS-NS and PANSS-WIT in DS, but not in NDS. ALFF in the cerebellum posterior lobe negatively correlated with PANSS-PS and PANSS-EXC/ACT in NDS, but not in DS. ALFF in the insula was positively associated with S2-P50 amplitude of sensory gating P50 in DS and HC, but not in NDS. ALFF in middle temporal gyrus was negatively correlated with P3b in NDS and HC, not in DS.		
**Functional MRI studies, task activation fMRI**											
**Author**	**Year**	***n* HC/DS/NDS**	**Findings with direct comparisons between HC, DS, and NDS**				**Associations with symptoms**				
Mucci et al.^55^	2015	22/11/17	DS = NDS = HC in activity in ventral striatum for the contrast reward v. neutral (during reward anticipation) DS < (HC = NDS) in activity in dorsal caudate for the contrast reward v. neutral (during reward anticipation)				Activity in dorsal caudate was negatively correlated with avolition, as measured by the SDS. Motivation, as measured by the SDS, was positively correlated with activity in dorsal caudate and ventral striatum				
Teles et al.^47^	2024	23.8 (5.3)/24.1 (5.6)/24 (6.5)	61/94/55	SDS	FE	Medication naïve	Whole brain	Graph theory	Global efficiency, local efficiency, shortest path length, synchronization, small-worldness, clustering coefficients	3	Open
King et al.^48^	2022	24 (5.7)/23.1 (5.2)/24 (6.3)	60.3/92/56.6	SDS	FE	Medication naïve	Frontoparietal control network	Seed-based and ROI	FC	3	Open
Fan et al.^49^	2022	45.9 (9.4)/48.7 (7.6)/46 (5.3)	100/100/100	SDS^a^	Chronic	Medicated	Whole brain	Graph theory	Intramodule connectivity, intermodule connectivity	3	Closed
Zhou et al.^52^	2021	50.8 (7.7)/51.5 (9.9)/51.3 (8.8)	100/100/100	SDS^a^	Chronic	Medicated	Nucleus accumbens	Seed based	FC	3	Closed
Zhou et al.^51^	2019	45.9 (9.4)/48.7 (7.6)/46 (5.3)	100/100/100	SDS^a^	Chronic	Medicated	Whole brain	ALFF	z-scored fALFF	3	Closed
Zhou et al.^50^	2019	46.1 (9.1)/49 (7.4)/45.8 (5.5)	100/100/100	SDS^a^	Chronic	Medicated	DMN	ICA	DMN	3	Closed
Yu et al.^53^	2017	45.9 (9.4)/48.7 (7.6)/46 (5.3)	100/100/100	SDS^a^	Chronic	Medicated	Whole brain	Graph theory	clustering coefficient, characteristic path length, normalized clustering coefficient, normalized characteristic path length, small-worldness, global efficiency, local efficiency	3	Closed
Li et al.^54^	2017	23.2 (7.3)/23.3 (6.9)/22.8 (6.7)	57/56/59	SDS	FE	Medication naïve	Whole brain	ALFF	fALFF	3	Closed
**Task activation fMRI**											
**Author**	**Year**	**Age HC/DS/NDS; mean (SD)**	**Sex HC/DS/NDS; % male**	**How DS is defined**	**Illness duration**	**Medication status**	**Regions of interest**	**Modality**	**Task**	**Eyes open/closed**	
Mucci et al.^55^	2015	HC: 31.91 (8.49), patients: 33.10 (6.67)	HC: 45, patients: 64	SDS	Chronic	Medicated	Ventral striatum, dorsal caudate	fMRI 3 T	Monetary incentive delay	Open	

^a^Chinese version

ACC = anterior cingulate cortex; ALFF = amplitude of low-frequency fluctuation; ANT = animal naming test; BPRS = brief psychiatric rating scale; CAL = calcarine sulcus; COWAT = Controlled Oral Word Association Test; DAN = dorsal attention network; DMN = default mode network; DS = deficit syndrome schizophrenia; FC = functional connectivity; FE = first episode patients; FPN = frontoparietal network; GMV = gray matter volume; HC = healthy controls; ICA = independent component analysis; IOG = inferior occipital gyrus; ITG = inferior temporal gyrus; LING = lingual gyrus; LNacc = left nucleus accumbens; MFG = middle frontal gyrus; N/A = not applicable; NDS = Non-deficit syndrome schizophrenia; OFC = orbitofrontal cortex; ORBinf = inferior orbitofrontal cortex; PANSS = Positive and Negative Syndrome Scale; PANSS-EXT/ACT = excited/activation score of the PANSS; PANSS-NS = negative symptom score of the PANSS; PANSS-PS = positive symptom score of the PANSS; PANSS-WIT = withdrawn score of the PANSS; PCC = posterior cingulate cortex; PFC = prefrontal cortex; PFC = prefrontal cortex; PHP / HIP = parahippocampal gyrus / hippocampus; rCBF = regional cerebral blood flow; RNAcc = right nucleus accumbens; SANS = scale for the assessment of negative symptoms; SD = standard deviation; SDS = Schedule for the Deficit Syndrome; TMT-B = trail-making test B; VFT = verbal fluency test; WAIS-RC = Wechsler Adult Intelligence Scale-Chinese Revision.

Findings that were replicated in at least 2 independent studies show that fractional anisotropy (FA), a non-specific measure of neuronal integrity, reductions are a shared pathology in the body of the corpus callosum,^41–43^ though another group reported FA reductions only in the splenium of the corpus callosum in patients who meet criteria for the deficit syndrome.^39^ In addition, FA was uniquely reduced in the uncinate fasciculus in deficit syndrome patients in region-of-interest analyses.^33^^,^^44^ However, uncinate fasciculus alterations were not detected in whole-brain white matter studies.

One study supporting the idea of the deficit syndrome being a more severe form of the illness found FA reductions are in the posterior thalamic radiation in both groups, though to a greater extent in the deficit syndrome.^41^ Another reported that FA reductions in the forceps major, the inferior-fronto-occipital fasciculus, and cerebellum, while present in both groups, are more prominent in patients who do not meet criteria for the deficit syndrome,^43^ though the same group also found the same extent of FA reductions in the corona radiata, forceps minor, external capsule, and cerebral peduncle in both subtypes.

Some studies directly contradict each other. While 2 studies found that lower FA in the superior longitudinal fasciculus was a shared feature across the subtypes,^25^^,^^40^ a relatively small study reported evidence of reduced FA only in the deficit subtype of the illness,^45^ and another detected increased radial diffusivity (RD) in this tract in both patient groups, but without corresponding FA reductions.^43^ Discrepancies in investigations in the superior longitudinal fasciculus may be due to differences in illness duration, medication status, the use of a region-of-interest vs a whole brain analysis, and how the deficit syndrome is defined, i.e., by the Schedule for the Deficit Syndrome (SDS) or by the Proxy for the Deficit Syndrome (PDS).

Finally, a limited number of studies also quantified white matter volumes, as well as mean diffusivity (MD), axial diffusivity (AD), or RD. These studies are in disagreement as to whether white matter abnormalities are unique to either subtype,^25^^,^^33^^,^^43^ are shared across the 2 subtypes,^39^^,^^43^^,^^46^ or support the idea of a continuum in severity.^43^

### Functional MRI Studies

Nine reports assessing functional brain indices met eligibility criteria for inclusion in the review. Table 3 summarizes the extracted data. Though not always explicitly stated, several reports appear to be based on overlapping or identical samples 1.^47^^,^^48^ and 2.^49–53^ All but one of the included studies used resting-state fMRI, and analysis methods varied widely, spanning seed-based, region of interest (ROI), fractional amplitude of low frequency fluctuations (fALFF), graph theory, and independent component analyses. One study used a monetary incentive delay task to assess differences in brain activation patterns.

**Table 3:** Magnetic Resonance Spectroscopy (MRS) Studies

**Author**	**Year**	***n* HC/DS/NDS**	**Age HC/DS/NDS; mean (SD)**	**Sex HC/DS/NDS; % male**	**Findings with direct comparisons between HC, DS, and NDS**	**Associations with symptoms**			
Bryant et al.^56^	2021	59 / 22 / 39	24.1 (5.7) / 23.2 (5.5) / 24.2 (6.5)	64 / 91 / 51	(DS = HC) < NDS in glutamate	None			
					DS = NDS = HC in NAA, Cr, Cho				
					HC = DS < NDS in mI				
Rowland et al.^45^	2009	11 / 10 / 10	37 (10) / 43 (6) / 40 (6)	72 / 80 / 80	DS = NDS = HC in NAA, Glx, Cho, Cr	None			
**Author**	**Year**	**How DS is defined**	**Illness duration**	**Medication status**	**Regions of interest**	**Metabolites**	**Tesla**	**MRS type**	**Sequence**
Bryant et al.^56^	2021	SDS	FE	Medication naive	Left frontal white matter	NAA, Glu, Cho, Cr, mI	3	SV	PRESS
Rowland et al.^45^	2009	SDS	Chronic	Medicated	IPC, left middle frontal cortex	NAA, Glx, Cho, Cr	3	SV	PRESS

Cho = choline; Cr = creatine; DS = deficit syndrome schizophrenia; FE = first episode patients; Glu = glutamate; Glx = glutamate-glutamine; HC = healthy controls; IPC = inferior parietal cortex; mI = myoinositol; MRS = magnetic resonance spectroscopy; NAA = N-acetyl-aspartate; NDS = Non-deficit syndrome schizophrenia; PRESS = point-resolved spectroscopy; SD = standard deviation; SDS = Schedule for the Deficit Syndrome; SV = single voxel.

#### Resting-State fMRI

Two findings were replicated in 2 independent samples: first, small-world properties in overall brain network organization were preserved in both patient groups^47^^,^^53^; second, disturbances in global efficiency and shortest path-length were identified as unique features associated with the deficit syndrome.^47^^,^^53^

Functional connectivity studies examining large-scale functional brain networks suggest that decreased connectivity in a subset of frontoparietal network regions^48^ and increased connectivity in the posterior cingulate cortex, a hub region of the default mode network,^50^ have been identified as unique features that are only present in patients who meet criteria for the deficit syndrome. The only fALFF abnormality unique to the deficit syndrome was reported in the hippocampus.^51^

Dysconnectivity in other default mode network regions^50^ and nucleus accumbens appears to be a shared feature among the 2 clinical subgroups.^52^ Similarly, fALFF alterations were detected in both clinical subgroups; the magnitude of alterations in cortical regions was comparable in both subgroups,^51^ while abnormalities in the putamen and cerebellum appear to differ in the degree of severity between the groups.^54^ Lastly, results for local network efficiency were contradictory, reporting reduced local efficiency to be a unique pathological signature in first episode patients presenting with clinical features of the deficit syndrome,^47^ and the same degree of local efficiency reductions in both clinical subgroups in the chronically ill patients.^53^ These conflicting findings could be associated with differences in illness chronicity and medication status.

#### Task fMRI

One investigation used a monetary incentive delay task to assess differences in ventral striatum and dorsal caudate activation patterns.^55^ They found that only patients with the deficit syndrome presented dorsal caudate hypoactivation for the neutral vs reward conditions. Ventral striatum activation was similar between patient subtypes and HC.

### MR Spectroscopy Studies

Two proton MRS studies met inclusion criteria for this systematic review.^45^^,^^56^ The first found no neurometabolite alterations in the left inferior parietal and left middle frontal cortices in either patient subtype.^45^ Our group investigated neurometabolites in frontal white matter in antipsychotic medication-naïve first episode psychosis (FEP) patients who presented with or without clinical features of the deficit syndrome.^56^ We found that frontal white matter glutamate was uniquely elevated in those who did not meet criteria for the deficit syndrome, while N-acetyl-aspartate (NAA), choline, and creatine did not differ between the 3 groups.

## Discussion

The deficit syndrome is conceptualized as a separate disease entity within the schizophrenia syndrome that is less heterogeneous, neurodevelopmental in its origin, characterized by fronto-parietal involvement and a lack of ventricle enlargement.^57^ In this systematic review, we identified nucleus accumbens and cerebellar gray matter volume deficits, reduced uncinate fasciculus FA, dysconnectivity in a subset of fronto-parietal and default mode network regions, as well altered global efficiency and shortest path-length in brain network organization as potentially unique features of the deficit syndrome, while increased hippocampus fALFFs and elevated white matter glutamate levels may be unique features of the non-deficit form of the illness. In support of the idea that the deficit syndrome is a more severe form of the same illness, others reported reductions in cerebellar culmen volume and posterior thalamic radiation FA, as well as an increase in putamen and cerebellum fALLFs in the deficit syndrome compared to the non-deficit form of the illness. However, many of these findings have not been replicated, and as such must be considered preliminary. For a summary of the main findings, see Table 4.

**Table 4:** Summary of Main Findings

**Unique to deficit syndrome**
Nucleus accumbens and cerebellar gray matter volume deficits
Lower uncinate fasciculus fractional anisotropy (FA)
Fronto-parietal and default mode network dysconnectivity
Altered global efficiency and shortest path-length in brain network organization
**Unique to non-deficit syndrome**
Higher hippocampus fractional amplitude of low frequency fluctuations (fALFFs)
Higher frontal white matter glutamate
**Supportive of the deficit syndrome as a more severe, i.e., not a distinct, form of schizophrenia**
More severe reduction in cerebellar culmen volume
Greater reductions in posterior thalamic radiation FA
More severe increase in putamen and cerebellum fALLFs

Currently, the deficit syndrome is hypothesized to have a neurodevelopmental origin, a unique fronto-parietal involvement, and a lack of ventricle enlargement; therefore, the following sections in the discussion will expand on how the MRI literature informs us of these common propositions regarding the deficit syndrome of schizophrenia.

### The Deficit Syndrome as a Neurodevelopmental Disorder

The deficit syndrome is thought to have a neurodevelopmental origin, with an early onset and a non-progressive nature, whereas the non-deficit form of the illness may result from attenuated brain plasticity in the context of neurotransmitter alterations. One study included in the review found reduced interthalamic adhesion length and altered subgyral patterns of the orbitofrontal cortex in the deficit form of the illness.^29^ Another report with a largely overlapping sample indicated that the odds of a Heschel gyrus duplication were approximately 4 times more likely in the deficit group compared to the non-deficit group,^58^ but this report did not meet inclusion criteria as not all 3 groups were considered in the same statistical model. The same group also contrasted cortical gyrification, a marker of early neurodevelopment, between patients who do or do not meet criteria for the deficit syndrome, in a presumably substantially similar sample.^59^ The authors report that the overall patient group had a widespread increase in cortical gyrification compared to HC. It is, however, difficult to clearly ascertain if only 1 patient group has this abnormality or if the subtypes only differ in degree of severity in hypergyrification, given the lack of comparisons across the 3 groups. Together, the extant literature of neuroimaging proxy markers of neurodevelopment consists of a single, relatively small sample where clinical subtyping was reliant on the proxy of the deficit syndrome, which is not the gold standard assessment. They found that some, but not all, neurodevelopment markers are unique to the deficit form of the illness. As such, there is insufficient neuroimaging evidence to support or refute the idea that the deficit syndrome is a neurodevelopmental disorder.

### Fronto-Parietal Involvement in the Deficit Syndrome

Patients with deficit syndrome are thought to present with distinctive fronto-parietal abnormalities; the current literature seems to suggest that such a fronto-parietal involvement may happen more so functionally than structurally in the brain. Structural neuroimaging studies found that cortical thinning in fronto-parietal brain regions^33^ and gray matter volume loss in the dorsolateral prefrontal cortex^31^ are common features across the clinical subtypes. Recently, the ENIGMA schizophrenia workgroup used structural neuroimaging data from 8 participating sites to investigate cortical thickness correlates of the deficit syndrome, based on the PDS.^60^ Using both meta- and mega-analytic methods to pool data, they found that cortical thinning was evident in both clinical subtypes. Furthermore, they noted that right hemisphere fronto-parietal cortical thinning was present only in the deficit syndrome, while left hemisphere fronto-parietal was more prominent in the deficit group but also present in the non-deficit form of the disease. In the superior longitudinal fasciculus, the major white matter tract that structurally connects the dorsolateral prefrontal and inferior parietal cortices, studies were consistent in finding that white matter integrity deficits are similar^40^^,^^42^^,^^43^ in both clinical subtypes of the illness, 1 reported that they are more prominent in the deficit syndrome.^45^

Functional imaging studies report that reduced within-network fronto-parietal connectivity in an ROI study,^48^ and increased intermodule connectivity between the fronto-parietal network and somatomotor^49^ are unique features of the deficit syndrome. In contrast, reduced fronto-parietal fALLFs were present in both subtypes of the illness in 1 study,^51^ but these abnormalities were not detected in either group in another study investigating fALLFs.^54^

Finally, neither of the 2 MR spectroscopy studies in frontal and parietal brain regions reported neurometabolite alterations in the deficit syndrome.^45^^,^^56^ Together, findings suggest that structural fronto-parietal alterations represent a shared biological feature in schizophrenia, whereas frontoparietal network dysconnectivity may be unique to the deficit syndrome.

### Ventricle Enlargement in the Deficit Syndrome

Interestingly, a lack of ventricle enlargement, hypothesized to be a hallmark feature distinguishing the 2 subtypes, was only reported in 1 of 2 independent studies^32^ that met our inclusion criteria. Another study that is often cited in support of this hypothesis compared patients with the deficit syndrome and HC, but did not actually compare ventricle volumes between groups, but rather total cerebrospinal fluid volume, which they found to be only numerically but not statistically significantly increased.^61^ The only other study investigating ventricle volumes in the context of the deficit syndrome used a clustering technique to identify neuroanatomical subtypes of schizophrenia, and found that patients with the biological subtype that includes ventricular enlargement are more likely to have the deficit syndrome than the other neuroanatomical subgroup.^62^ We also note that reduced thalamus, insula, and striatum volumes were identified as major contributors to ventricle enlargement in schizophrenia,^63^ and none of the studies included here identified these as unique features of the deficit form of the illness. Together, existing data show that ventricle volumes may not biologically differentiate the 2 clinical subtypes of the illness.

### Limitations

There are limitations that need to be acknowledged. While we identified a considerable number of MRI manuscripts that investigated neural substrates of the deficit syndrome, many of them appear to be based on overlapping samples. It is important to note that the value of confirming a finding in a substantially similar sample should not be considered a replication. We also excluded MRI findings in the deficit syndrome that did not directly compare the 2 patient subgroups and HC in the same statistical model. We made this choice because, without a “normal” reference cohort, it is difficult to ascertain if findings differentiating the 2 subtypes actually fall within the range of normal and, as such, may not reflect a pathological finding. Furthermore, most studies had uneven sample sizes and sex distributions in their samples. It is unclear if assumptions of homogeneity in variance in subgroups were violated, which could lead to reduced statistical power to detect effects. We considered meta-analyses to mitigate this issue. However, guidelines on neuroimaging meta-analyses recommend having at least 17-20 experiments included to detect small to medium effect sizes,^64^ and that ROI data should not be included because it would lead to inflated significance for those regions that come from overrepresented ROI analyses.^65^ As such, we chose a qualitative rather than quantitative approach. Additionally, a few studies^29^^,^^33^^,^^39–41^ employed the PDS as the method for patient group categorization, as opposed to the SDS. While this may add heterogeneity to the findings of this review, the PDS was demonstrated as a reliable and accurate method for the identification of the deficit syndrome.^66^

## Conclusions

The central theorem posits that the deficit syndrome is different from other forms of the illness and more homogenous in its pathophysiology. There is clinical and epidemiological data to support this. However, it is important to note that neuroimaging studies thus far have only examined group differences in brain signatures with conflicting results, but none have tested if inter-individual heterogeneity in brain pathology actually differs between the 2 clinical subtypes of the schizophrenia syndrome. This is perhaps not surprising, because until recently, statistical methods allowing to assess brain pathology at the individual patient level did not exist. With the advent of normative modeling for the brain,^67^ it is now possible to more directly test if this clinically more homogenous subtype of the illness is also biologically less heterogeneous. If this hypothesis can be empirically confirmed, there will be an opportunity to potentially close the translational gap and create the mechanistic framework necessary to develop specific and personalized treatments for patients suffering from the deficit syndrome.
